# Opportunities, Challenges, and Lessons Learned From Partograph Utilization for Labor Monitoring in Sub-Saharan Africa: A Systematic Review

**DOI:** 10.7759/cureus.82295

**Published:** 2025-04-15

**Authors:** Godfrey R Mugyenyi, Wilson Tumuhimbise, Joseph M Ntayi, Josaphat K Byamugisha, Angella Musimenta, Musa Kayondo, Joseph Ngonzi, Yarine Tornes, Esther C Atukunda

**Affiliations:** 1 Obstetrics and Gynecology, Mbarara University of Science and Technology, Mbarara, UGA; 2 Computing and Informatics, Mbarara University of Science and Technology, Mbarara, UGA; 3 Procurement, Logistics, and Supply Management, Makerere University Business School, Kampala, UGA; 4 Obstetrics and Gynecology, Makerere University College of Health Sciences, Kampala, UGA; 5 Pharmacy, Mbarara University of Science and Technology, Mbarara, UGA

**Keywords:** challenges, labor monitoring, opportunities, partograph, systematic review, utilization

## Abstract

Despite decades of healthcare provider training and investment, rates of utilization and capacity to appropriately plot the partograph and use it to make critical decisions remain sub-optimal in Sub-Saharan Africa. The objective of this review was to synthesize evidence of existing challenges and opportunities of partograph utilization in labor monitoring in Sub-Saharan Africa from existing literature. PUBMED, Cochrane Library of Trials, Google Scholar, and ScienceDirect databases published between January 1, 2013, and July 31, 2023, for studies reporting opportunities and challenges of utilizing the partograph in labor monitoring in Sub-Saharan Africa, were searched. Preferred Reporting Items for Systematic Reviews and Meta-Analyses guided the identification of eligible studies. We utilized a Consolidated Framework for Implementation Research to interpret findings. The review protocol was registered under PROSPERO-CRD42023452882. Twenty-eight high-quality studies identified from a total of 452 in eight countries in Sub-Saharan Africa were analyzed in this review. Opportunities identified included 1) the partograph’s uncontested potential to reduce maternal and neonatal mortality facilitated by good support supervision, formal and on-the-job training, active mentorships, and ongoing supervision, and the availability of enabling policies, standards, and protocols on partograph use; 2) the availability of partographs in health facilities. Gaps and challenges identified included 1) lack of inadequate training and tool complexity, 2) availability of different labor monitoring tools other than the partograph, 3) lack of motivation and feedback, 4) unavailability of the partograph in some health facilities, 5) shortage of staff in busy facilities, 6) lack of support supervision, 7) lack of protocols, 8) inappropriate motivation leading to 9) lack of commitment, negative attitude, negligence, careless partograph completion, or non-use. The challenges, opportunities, and lessons learned from this review on partograph utilization in labor monitoring will help develop suitable implementation strategies to guide the introduction and scale-up of the labor care guide in Sub-Saharan Africa if we are to avoid similar challenges.

## Introduction and background

The partograph consists of several key components, including maternal observations (such as pulse, blood pressure, temperature, and contractions), fetal observations (such as fetal heart rate and amniotic fluid status), and labor progress indicators (such as cervical dilation and fetal descent). To enhance its utility, Philpott and Castle introduced the alert and action lines in 1972, creating a pictorial, paper-based overview that allows healthcare providers to identify deviations from normal labor and intervene early [1]. Originally proposed by Friedman in 1955 as a universal tool for monitoring labor to prevent prolonged and obstructed labor, the partograph became the gold standard for labor monitoring [2]. Since its introduction, the original partograph has undergone several improvements and modifications to address challenges such as inadequate human resources and training [3,4]. This partograph has different sections, including a record of patient biodata (identifying the mother, including risk assessment and critical labor events), a section for recording assessments of the fetal condition, including fetal heart rate and status of membranes, a record of maternal condition, and finally a section for recording the progress of labor [1-4].

Over time, the partograph has demonstrated benefits in averting prolonged and obstructed labor and has also been documented as an inexpensive and practical tool for differentiating abnormal from normal labor progress, thereby reducing operative deliveries and improving birth outcomes in low-, middle-, and high-income countries [1,5]. Despite decades of healthcare provider training and investment in human resources, rates of utilization and capacity to appropriately plot the partograph and use it to make critical decisions remain sub-optimal, specifically in low- to middle-income countries [6-8], with correspondingly high incidences of obstructed labor and its associated maternal and neonatal mortalities [9-11]. Other scholars have also noted that the partogram offers subjective variations that assume that all women progress at the same rate and thus affect intervention rate and individualized care [12]. In Uganda, the partograph was found to be commonly used to record birth outcomes and not the actual monitoring of labor for which it is intended [13].

A review by Lavender and colleagues documented an insignificant effect of the partograph on labor monitoring [8]. Studies suggest that there could be wider challenges that may be hindering the optimal and effective utilization of this tool, which could range from intervention/tool and individual characteristics to outer and inner settings, as well as the implementation process. Although two reviews [14,15] have documented the challenges of utilizing the partograph and factors contributing to the successes or failures of the partograph in labor monitoring in low- and middle-income countries, there has not been any review specific to Sub-Saharan Africa, where the burden of prolonged/obstructed labor is high, contributing to 2-8% of maternal deaths, while almost non-existent in high-income countries [16,17]. In Uganda, over 8% of all maternal deaths were linked to obstructed labor, with over 90% of perinatal mortality following birth asphyxia directly attributed to obstructed labor [18]. There is, therefore, a need to comprehensively synthesize gaps/challenges and opportunities peculiar to Sub-Saharan Africa to inform stakeholders, policymakers, and researchers amidst the WHO's call to take on new, effective interventions such as the labor care guide.

According to Vogel and others, the labor care guide is regarded as the “next generation” partograph, with modifications to improve labor monitoring and other delivery outcomes [19]. This is achieved through encouraging/initiating or stimulating ongoing shared decision-making among health workers, women, and labor companions to promote woman-centered care and provide timely decision-making [20] while offering each woman a positive pregnancy and childbirth experience [21,22]. In this review, we utilize the Consolidated Framework for Implementation Research (CFIR) [23] to synthesize the challenges/gaps of labor monitoring while using the partograph and use the lessons learned to inform stakeholders engaged in designing suitable implementation approaches for the new labor monitoring tools in low-resource settings.

This paper was uploaded as a preprint to Research Square on October 5, 2023.

## Review

Methods

Overview of Methods for This Review

We conducted this review using a stepwise guide for researchers engaged in systematic reviews within the field of medicine and health, as described by Khan and colleagues in 2003 [24] and modified by Calderon Martinez et al. in 2023 [25]. They described a series of structured steps designed to ensure the rigor and reliability of the review process, including 1) framing a clear, focused research question(s); 2) identifying relevant work; 3) assessing the quality of studies; 4) summarizing the evidence; and 5) interpreting the findings. We developed the review protocol with a detailed plan outlining the methods and criteria for this review. We registered the review protocol in PROSPERO under registration number CRD42023452882 on August 21, 2023, accessible at crd.york.ac.uk/PROSPERO/display_record.php?RecordID=452882. The authors ensured a comprehensive search of relevant databases, including PUBMED, Cochrane Library of Trials, Google Scholar, and ScienceDirect, to identify all relevant studies using a combination of keywords, Medical Subject Heading (MeSH) terms, and Boolean operators. We screened all identified studies based on predefined inclusion and exclusion criteria at the title/abstract, followed by full-text screening to select high-quality studies for full review. We extracted and tabulated relevant data from the selected studies using standardized forms, including authors and year of publication, location of the study by country, study design, interventions, outcomes, and results, capturing challenges/gaps and opportunities to partograph utilization in Sub-Saharan Africa published from July 2013 to July 2023. Each identified study was assessed for quality [26], with subsequent data extraction, synthesis, and contextualization in line with predetermined research questions using CFIR to draw conclusions and recommendations for practice and future research. This review utilized five CFIR domains [23], including intervention characteristics, inner setting, outer setting, intervention characteristics, and implementation process, to systematically synthesize the partograph challenges/gaps and derive lessons to guide effective labor monitoring using new tools. Review results are reported using the Preferred Reporting Items for Systematic Reviews and Meta-Analyses (PRISMA) [27].

Identification of Studies

**Figure 1:**
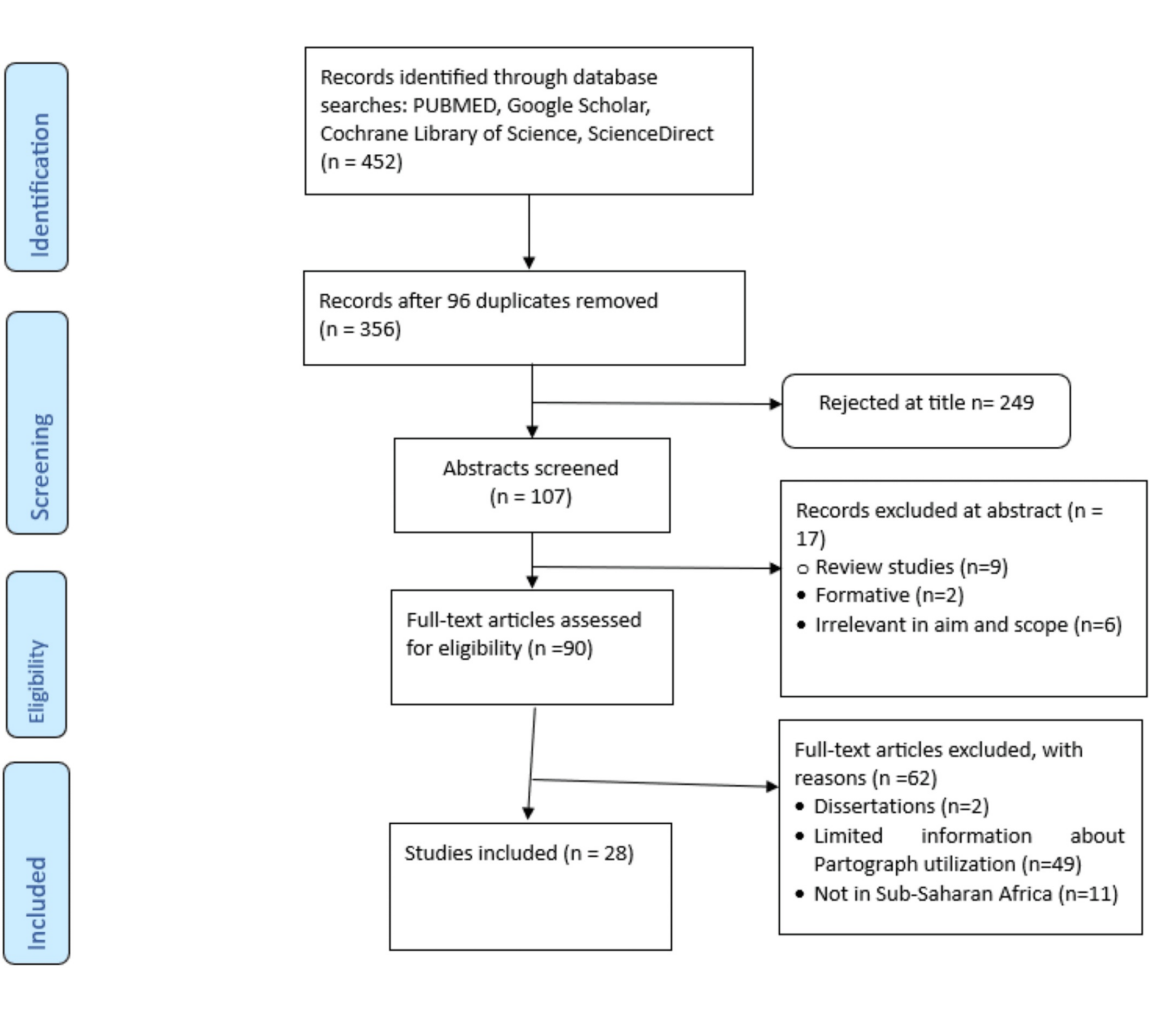
PRISMA flow diagram for selected studies PRISMA: Preferred Reporting Items for Systematic Reviews and Meta-Analyses

A comprehensive search strategy was conducted in August 2023 to identify studies documenting the challenges and opportunities of partograph use in labor care monitoring in Sub-Saharan Africa for inclusion. The identification of the eligible studies for inclusion in this systematic review is presented in the PRISMA flowchart (Figure 1). The authors reviewed articles that were published between January 1, 2013, and July 31, 2023, to understand the most recent literature about existing opportunities, gaps, and challenges of partograph utilization for labor monitoring. The following research questions guided our search for this systematic review: (i) What facilitates the utilization of the partograph in labor monitoring in Sub-Saharan Africa? (ii) What hinders the utilization of the partograph in labor monitoring in Sub-Saharan Africa? Therefore, the articles identified by the reviewers intended to address the questions above.

Search Strategy

PUBMED, Cochrane Library of Trials, Google Scholar, and ScienceDirect bibliographical databases were searched in August 2023 for peer-reviewed articles following key search terms to denote "partograph utilization", "challenges", "opportunities", and "Labor monitoring". Each word was entered as a keyword in the bibliographical databases to identify the relevant articles. The bibliography/reference lists of the included studies were also screened to get more studies for inclusion in the study. To capture research articles that were related to each concept, we used a Boolean search using the union ("OR") and the intersection ("AND") of the key concepts to focus on the main objective of this review (Table 1). Results obtained during the search query were uploaded in the EndNote X7 Reference Manager (Thomson Reuters, Philadelphia, PA, USA), where they were independently assessed for inclusion based on title, abstract, and full text. The decision regarding the inclusion and exclusion of studies was discussed among the authors until a general consensus was reached.

**Table 1:** Search strategy for partogram utilization in labor monitoring in Sub-Saharan Africa

Database	Search query	Results
ScienceDirect	'Partograph utilization' OR 'partogram utilization' AND 'Challenges' in Labour monitoring in Low resource	44 results
Google Scholar	"partograph" OR "partogram" AND "Challenges" AND "Opportunities" AND "Low resource"	147 results
Cochrane Library	(partograph) OR (partogram) AND (utilization) AND (labour) AND (Low resource)	129 trials, 6 reviews
PubMed	(((partograph) OR (partogram)) AND (utilization)) AND (labour)	99 results
Detailed search	(((partograph) OR (partogram)) AND (utilization)) AND (labour) Filters: Free full text, English, from 2013 - 2023	Various results
Search strategy	("partograph"[All Fields] OR "partographic"[All Fields] OR "partographs"[All Fields] OR ("partogram"[All Fields] OR "partograms"[All Fields])) AND ("statistics and numerical data"[MeSH Subheading] OR ("statistics"[All Fields] AND "numerical"[All Fields] AND "data"[All Fields]) OR "statistics and numerical data"[All Fields] OR "utilization"[All Fields] OR "utilisation"[All Fields] OR "utilisations"[All Fields] OR "utilise"[All Fields] OR "utilised"[All Fields] OR "utilises"[All Fields] OR "utilising"[All Fields] OR "utilities"[All Fields] OR "utility"[All Fields] OR "utilizations"[All Fields] OR "utilize"[All Fields] OR "utilized"[All Fields] OR "utilizer"[All Fields] OR "utilizers"[All Fields] OR "utilizes"[All Fields] OR "utilizing"[All Fields]) AND ("labor s"[All Fields] OR "labored"[All Fields] OR "laborer"[All Fields] OR "laborer s"[All Fields] OR "laborers"[All Fields] OR "laboring"[All Fields] OR "labors"[All Fields] OR "labour"[All Fields] OR "work"[MeSH Terms] OR "work"[All Fields] OR "labor"[All Fields] OR "labor, obstetric"[MeSH Terms] OR ("labor"[All Fields] AND "obstetric"[All Fields]) OR "obstetric labor"[All Fields] OR "laboured"[All Fields] OR "labourer"[All Fields] OR "labourers"[All Fields] OR "labouring"[All Fields] OR "labours"[All Fields])) AND ((ffrft[Filter]) AND (english[Filter]) AND (2013:2023[pdat])	Detailed breakdown
Translations		
Term	Search Keywords	
Partograph	"partograph"[All Fields] OR "partographic"[All Fields] OR "partographs"[All Fields]	
Partogram	"partogram"[All Fields] OR "partograms"[All Fields]	
Utilization	"Statistics and numerical data"[Subheading] OR ("statistics"[All Fields] AND "numerical"[All Fields] AND "data"[All Fields]) OR "statistics and numerical data"[All Fields] OR "utilization"[All Fields] OR "utilisation"[All Fields] OR "utilisations"[All Fields] OR "utilize"[All Fields] OR "utilized"[All Fields] OR "utilizes"[All Fields] OR "utilizing"[All Fields]	
Labour	"labor's"[All Fields] OR "labored"[All Fields] OR "laborer"[All Fields] OR "laborer's"[All Fields] OR "laborers"[All Fields] OR "laboring"[All Fields] OR "labors"[All Fields] OR "labour"[All Fields] OR "work"[MeSH Terms] OR "work"[All Fields] OR "labor"[All Fields] OR "labor, obstetric"[MeSH Terms] OR ("labor"[All Fields] AND "obstetric"[All Fields]) OR "obstetric labor"[All Fields] OR "laboured"[All Fields] OR "labourer"[All Fields] OR "labourers"[All Fields] OR "labouring"[All Fields] OR "labours"[All Fields]	

Study Selection Criteria

The studies were included in the review if they met the following criteria: 1) peer-reviewed; 2) full research paper available; 3) both qualitative and quantitative research; 4) research methods were clearly explained; 5) study available in English language; 6) explicitly described opportunities and/or challenges of using the partograph for labor monitoring or the challenges and opportunities; 7) published between January 1, 2013, and July 31, 2023; and 8) conducted in Sub-Saharan Africa.

Criteria 1-5 were considered to ensure the reporting of original research and high-quality work. Criterion 6 was included to ensure that the paper reported the partograph utilization in labor monitoring. Our definition of partograph utilization opportunities included the advantages, benefits, facilitating factors, and motivators for using the partograph, while we defined the challenges as difficulties, barriers, gaps, and problems encountered while using the partograph for labor monitoring. Criterion 7 was considered because 2013 saw an unprecedented introduction of modifications to the partograph [28]; therefore, the inclusion of articles before 2013 would report findings that would not adequately capture the recent global improvements. Furthermore, in 2018, the WHO published a consolidated set of guidelines on intrapartum care for a positive childbirth experience that recommended the labor care guide as one of the evidence-based interventions with the potential to improve delivery outcomes. Criterion 8 was considered because the partograph was the main tool used in labor monitoring in Sub-Saharan Africa, a region that accounted for 70% of all maternal deaths (202,000 out of 287,000) in 2020 [29]. The new labor care guide, regarded as the “next-generation partograph,” has the potential to improve delivery outcomes in this region [7,30].

Exclusion Criteria

We excluded studies that did not report partograph utilization challenges or opportunities in labor monitoring, not carried out in Sub-Saharan Africa, carried out before 2013, and those that were not relevant to the objective of this review. We only included studies that documented reasons for utilizing and not utilizing the partograph. Those who discussed the partograph as a determinant of labor outcomes or recommended the partograph for improving labor outcomes were not considered, since we were interested in the actual experiences of using the partograph in labor monitoring. The studies that were considered were carefully examined to ensure that they reported the challenges and opportunities of partograph utilization; therefore, protocols, reviews, formative studies, editorials, letters, and position papers were not considered. All other studies were included irrespective of the study design.

Data Extraction

The following characteristics were extracted from the relevant included studies: authors, study setting, outcomes on labor monitoring, study design, challenges, opportunities, study duration, and measures of effect (including mean ratios, risk ratios, odds ratios, and confidence intervals) as shown in Table 2. The differences regarding the inclusion of studies were reviewed and checked by all authors to ascertain compliance with the inclusion criteria.

**Table 2:** Characteristics of the studies included in this review AOR: adjusted odds ratio; CI: confidence interval; OCP(s): obstetric care provider(s)

Study	Study design/method/location	Objective	Opportunities identified	Challenges identified
Yisma et al. 2013 [31]	A cross-sectional quantitative study that assessed knowledge and utilization of the partograph among 202 obstetric care givers in public health institutions of Addis Ababa, Ethiopia. Duration: 1 month	To find out obstetric care givers’ knowledge and use of the partograph in the public health institutions	Prevent prolonged labor and facilitate early referral to specialized health facilities (97.9%)	Little or no knowledge of the partograph (30.66%); Time consuming (28.23%); Lack of adequate number of personnel (6%); Lack of training (8.06%); Too much detail to fill (10.48%)
Wakgari et al. 2015 [9]	A cross-sectional study in North Shoa zone, central Ethiopia among 403 eligible obstetric care providers. Duration: 15 days	To assess the level of partograph utilization and its associated factors among obstetric care providers	Getting on job training (AOR = 2.86, 95% CI: 1.69, 4.86); Being knowledgeable on partograph (AOR = 3.79, 95% CI: 2.05, 7.03); Having a positive attitude towards partograph (AOR = 2.35, 95% CI: 1.14, 4.87)	Different monitoring tools other than partograph (52.6%); Shortage of staff (14.02%); Unavailability of the partograph (21.05%); Lack of trained human power (12.3%)
Wakgari et al. 2015 [32]	An institution-based cross-sectional study among 403 obstetric care providers. Duration: 1 month	To assess knowledge of partograph and its associated factors among obstetric care providers in North Shoa Zone, Central Ethiopia.	Received on job training (AOR = 5.49, P = 0.001, 95% CI: 3.32, 9.08)	Complexity of the partograph
Zelellw and Tegegne 2018 [33]	A cross-sectional mixed methods study in East Gojam Zone, Northwest Ethiopia. Duration: 5 months	To assess the usage and perceived barriers of obstetric caregivers at public health institutions	Partograph can reduce maternal and fetal mortality and morbidity, prevent prolonged and obstructed labor, and prevent postpartum hemorrhage.	Work overload; Skill incompetency and knowledge gaps; Negligence; Lack of motivation and a shortage of infrastructure and resources; Misperception
Bazirete et al. 2017 [34]	A descriptive quantitative and cross-sectional study among 131 nurses and midwives providing obstetric care in 15 health institutions in Rwanda	To describe factors affecting utilization of the partograph among nurses and midwives in selected health facilities of Rwanda	Training received in management of labor. Number of years of professional experience	Insufficient numbers of staff to monitor pregnant women in labor; Lack of in-service training on how to manage women in labor.
Lumadi 2017 [35]	A qualitative, exploratory and descriptive study among 17 healthcare workers at Vhembe District of Limpopo Province, South Africa	To explore and describe the perceptions of midwives on auditing of the partograph by health professionals and to explore and describe the perceptions of midwives on the feedback that was given after audit was done	Positive feedback on the use of the partograph	Lack of knowledge on the use of the partograph; Lack of encouragement and praise when documentation was done correctly and emphasis was mostly placed on negative aspects. Limited time and shortage of staff to provide timely, sufficient and specific feedback
Ayebare et al. 2020 [36]	A sequential explanatory mixed methods study which comprised of i) medical records review, ii) observation of monitoring practices during labor, iii) in-depth interviews and iv) focus group discussions with health workers in Northern Uganda. Duration: 1 year	To explore Fetal Heart Rate monitoring practices among health workers at a public hospital	Emotional consequences of failure to monitor the Fetal heart rate	Plotting the findings later may result in forgetting the findings and inaccurate recordings. Shortage of beds hinders the health workers’ ability to monitor. Women’s unwillingness to be examined frequently
Sama et al. 2017 [37]	Analytical cross-sectional study among 71 obstetric care providers in Cameroon. Duration: 3 months	To explore the current level of knowledge and factors associated with the use of the partograph among obstetric care providers (OCPs)	Reduce maternal morbidity (60.6%); Reduce maternal mortality (78.9%); Increase the efficiency of those attending to women in labor (64.8%).	Little or no knowledge on completing the partograph (35.4%); Shortage of staff (31.3%); Easier to manage labor without using of the partograph (8.3%)
Zelellw and Tegegne 2018 [12]	A cross-sectional study in East Gojam Zone, Northwest Ethiopia. Duration: 5 months	To assess the level of partograph use and its associated factors among obstetric caregivers	Comprehensive knowledge of the pantograph, on-the-job training in obstetric care	Lack of knowledge (30.95%); Much detail to fill (29.37%); Time consuming (20.63%); Lack of training (19.05%)
Okokon et al. 2014 [38]	A descriptive quantitative study among 290 nonphysician obstetric care workers in Calabar, Nigeria. Duration: 6 months	To determine and compare the factors that influence utilization of the partograph in primary, secondary, and tertiary health care delivery levels in Calabar, Nigeria	Detecting prolonged labor (71.0%); Detecting poor progress of labor (71.0%); Detecting insufficient uterine contractions (62.1%); Detecting suspected fetal distress (60.7%); Detecting need for labor augmentation (60.3%); Detecting abnormal fetal heart rate (58.6%); Detecting need for Cesarean delivery (57.2%); Detecting obstructed labor (56.6%)	Little or no knowledge (79.0%); Non-availability of the partograph (58.6%); Shortage of staff (46.9%); Time consuming (24.1%).
Bedada et al. 2020 [39]	Facility-based cross-sectional study among 325 OCPs in the West Shoa Zone, Central Ethiopia. Duration: 2 months	To assess the level of utilization of partograph and associated factors among obstetric care providers working at health facilities	Training (AOR = 3:94, 95% CI: 1.99-7.78); Availability of partograph (AOR = 5:23, 95% CI: 1.69-16.22); Perceived as not time-consuming task (AOR = 3:61, 95% CI: 1.19-10.96); Adequate number of obstetric care providers available (AOR = 2:92, 95% CI: 1.16-7.33); Supervision (AOR = 4:35, 95% CI: 2.11-8.97); Positive attitude (AOR = 2:48, 95% CI: 1.23-5.02); Availability of standard protocol in a health facility (AOR = 4:71, 95% CI: 2.31-9.60)	Partograph chart not available; Shortage of health care personnel; The absence of obligation from hospital/health center policy to perform; Lack of orientation/training on how to use (112, 34.8%); Availability of other methods of observation (43, 13.4%); Lack of commitment (128, 39.8%); Workload (199, 61.8%); Lack of supervision (172, 53.4%).
Jagau et al. 2017 [40]	A retrospective audit partograph assessment study that included the review of 538 partographs from Mowbray Maternity Hospital in Cape Town, South Africa. Duration: 5 months	To assess barriers and enablers for the use of a partograph in under-resourced settings, especially to monitor fetal heart rate.	Ability to safeguard healthy labor outcomes for both mother and child	Feeling unconfident to use the partograph; Busy to have time to write down observations; Lack of knowledge to use the partograph correctly; Lack of training to use the partograph
Ayehubizu et al. 2022 [41]	An institution based cross-sectional quantitative among 235 obstetric care givers in Somali region, Ethiopia. Duration: 2 months	To assess partograph utilization and associated factors among obstetric care givers in governmental health institutions of Jigjiga and Degehabur Towns, Somali Region, Ethiopia	Availability of pantograph (AOR = 4.633, 95% CI: (1.698–12.640)); Having good knowledge (AOR = 6.90, 95% CI (2.62–18.18)); Receiving on job training (AOR = 15.46, 95% CI: (6.95-34.42)); Positive attitude toward pantograph (AOR = 2.99, 95% CI: (1.25-7.14))	Lack of orientation (42.5%); Lack of commitment (26.8%); Lack of supervision (20.2%); Availability of other methods (10.5%)
Haile et al. 2020 [10]	A facility-based cross-sectional study among 436 health professionals in Hadiya Zone, Southern Ethiopia. Duration: 1 month	To determine partograph utilization and associated factors among obstetric care providers at public health facilities in Hadiya Zone, Southern Ethiopia	Received on-the-job training on partograph (AOR = 7:06; CI = 4:3, 11.37); Knowledgeable about partograph (AOR = 2:12; CI = 1:3, 3.9); Favorable attitude toward partograph use (AOR = 1:8; CI = 1:12-2:97)	Unavailability of partograph in the labor ward (31.86%); Absence of on-the-job training (29.92%); Lack of supervision (19.78%); Partograph is time-consuming (13.18%); Lack of trained human power (5%)
Melese et al. 2020 [42]	An institution-based cross-sectional survey among 459 obstetric care providers. Duration: 2 months	To assess the utilization of partograph and associated factors among obstetric care providers working in Wolaita zone health facilities, Southern Ethiopia	Having knowledge about the partograph Positive attitude towards the partograph	Utilization of different monitoring tools; Unavailability of the partograph
Markos et al. 2020 [43]	A health facility-based quantitative cross-sectional study among 269 obstetric caregivers. Duration: 3 months	To assess the magnitude of partograph utilization and factors that affect its utilization among obstetric caregivers in public health facilities of Wolaita Zone, Ethiopia	On-the-job training (AOR = 0:16, 95% CI: 0.06-0.43); Good knowledge (AOR = 3:35, 95% CI: 1.61-6.97); Favorable attitude towards partograph utilization (AOR = 2:99, 95% CI: 1.28-7.03)	Unavailability of the partograph; Lack of training; Lack of supervision; Time consuming; Using different tools; Workload; Absence of managerial body
Gebreslassie et al. 2019 [44]	A cross-sectional study among 406 obstetric care givers in the Eastern zone of Tigray. Duration: 2 months	To evaluate the utilization of the partograph and associated factors among obstetric care providers in the Eastern zone of Tigray, Northern Ethiopia	Having qualified from pre-service training (AOR = 0.01, 95% CI = (0.02, 0.05))	The higher the level of education, the lower the utilization of the partograph
Brits et al. 2020 [45]	A two-phase, quantitative, cross-sectional descriptive study design among 12 nursing personnel in South Africa	To assess the knowledge and attitudes of nursing personnel and to evaluate their practices of completing partographs at National District Hospital, South Africa.	Belief that partographs are useful and could help them to identify problems during labor (90%); Positive attitude toward the usefulness of partographs	Difficulty to divide their attention between the patient and the partograph. Shortages of personnel; Busy units force participants to fill in partographs after the labor, or they do not complete it at all; Lack of formal training on completing partographs
Yang et al. 2017 [46]	A qualitative study that involved 70 In depth interviews and 16 Focus group discussions in Federal Capital Territory (Abuja) and Ondo State, Nigeria, and Kampala, Uganda. Duration: 5 months	To explore current practices, challenges, and opportunities in relation to monitoring labor progression, from the perspectives of healthcare professionals in low-resource settings	The partograph considered as the most important tool for labor monitoring; Distinguishes the abnormal from the normal in labor progression; Enables decision-making; Passes health information among midwives when handing over shifts, and between midwives and doctors during reporting	Partograph were completed retrospectively after childbirth to fulfill hospital protocol requirements. Nonuse or incorrect use of the partograph Lacked knowledge to complete Overwhelming workload
Mezmur et al. 2017 [47]	An institution based cross-sectional quantitative study among 441 health professionals working in public health institutions in Ethiopia Duration: 2 months	To assess the knowledge and utilization of the partograph and associated factors among health professionals at public health institutions in eastern Ethiopia	In-service training (AOR = 2.0, 95% CI: 1.22, 3.42); Positive attitude about the partograph (AOR = 2.90, 95% CI: 1.30, 6.30)	Lack of training for health professionals; Negative attitude
Hagos et al. 2020 [48]	An institution based cross-sectional study design among 605 midwives in Addis Ababa, Ethiopia. Duration: 2 months	To assess utilization of partograph and its predictors among midwives working in public health facilities, Addis Ababa city administration, Ethiopia	Being mentored (AOR = 3.1; 95% CI: 1.7, 5.3); Received training (AOR = 2.4; 95% CI:1.5, 3.6); Being knowledgeable about partograph (AOR = 1.6; 95% CI: 1.1, 2.5); Supportive supervision 4 times per year (AOR = 18.6; 95% CI: 6.6, 25)	Lack of commitment (348 (58.6%)); Lack of supervision (121 (20.4%)); Lack of training (99 (16.7%))
Tilahun et al. 2021 [49]	An institutional-based cross-sectional study among 393 obstetric caregivers in Southwest Ethiopia. Duration: 4 months	To investigate the utilization of partograph and associated factors among obstetric caregivers in public health institutions of Southwest Ethiopia	Trained on partograph use (AOR = 7.83 (95% CI: (4.54, 13.50)); Good knowledge about partograph (AOR = 5.84 (95% CI: (3.27, 10.44))	Unavailability of partograph (84 (37.5%)); Lack of training on how to use partograph (46 (20.53%)); Partograph time consuming (27 (12.05%)); Shortage of staff (32 (14.28%)); Easier to use another monitoring tool (17 (7.58%)); The higher the level of education, the lower the utilization
Negash and Alelgn 2022 [50]	An institution based cross-sectional study among 370 skilled delivery providers of public health facilities of Hawassa city, Sidama region. Duration: 1 month	To assess magnitude and factors associated with proper partograph recording among skilled delivery attendants in public health facilities of Hawassa city, Sidama, Ethiopia	On job training (AOR = 1.9, 95% CI: (1.1, 3.2)); Good knowledge (AOR = 3.1, 95% CI: (1.8, 5.3); Supportive supervision (AOR = 4.5, 95% CI, 2.5, 7.9)	Lack of knowledge
Mukisa et al. 2019 [51]	A qualitative and hospital-based cross-sectional descriptive chart review at Mulago National Referral Hospital, Kampala, Uganda. 32 healthcare workers. Duration: 3 months	To determine the level of Partograph completion and healthcare workers perspectives towards its utilization.	Partograph important for handover of care at shift change.	The availability of other monitoring tools; Limitation in skills Inadequate equipment and supplies; High workload; The state of the mother at the presentation to the hospital; Lack of availability of the partographs
Hailu et al. 2018 [52]	Facility based cross sectional study among 220 obstetric care givers in in central zone, Tigray, Ethiopia. Duration: 7 months	To assess partograph utilization and associated factors among obstetric care givers	Favorable attitude towards partograph utilization 134 (67.7%); Using the partograph decreases risks of mother/infant morbidity and mortality 197 (99.5%); Basic emergency obstetric and new born care training	Being young in age were less likely to use the partograph than those older; Male obstetric caregivers were also 63% less likely to use the partograph (AOR (95% CI) 0.37 (0.44–0.95))
Anokye et al. 2019 [53]	A retrospective study design using a quantitative approach that reviewed 200 patient folders in the Volta Region of Ghana. Duration: 1 year	To assess the impact of the use and completion of partograph during labor on reducing birth asphyxia at the St. Anthony’s Hospital, Dzodze	Partograph utilization associated with less non-asphyxiated birth outcomes.	Inadequate documentation of the parameters monitored
Konlan et al. 2016 [54]	A cross-sectional descriptive study among 140 midwives in Tamale Metropolis, Ghana. Duration: 5 months	To assess the knowledge level of midwives on the effective use of the partograph in monitoring the progress of labor in the Tamale Metropolis of Ghana	Availability of the partograph sheets in the health facilities; In-service training on the use of the partograph	Inadequate knowledge on the proper use of the tool; Inadequate staffing; Extra workload on the few midwives
Asibong et al. 2014 [55]	A cross-sectional descriptive study among 130 Obstetrics care givers working in the General Hospital, Calabar, Nigeria	To evaluate the non-physician obstetric caregivers’ (OCGs) knowledge of partograph use, assess the extent of its use, determine the factors that impede its usage, and unravel the relationship between years of experience and partograph use among the respondents (OCGs) in General Hospital, Calabar, Nigeria	Detecting prolonged labor (73.1%); Obstructed labor (detection 64.6%); Poor progress of labor (80.8%); Detection of Inefficient uterine contraction (68.5%); Identification of abnormal fetal heart rate (66.9%)	Little or no knowledge of the partograph (85.4%); Non-availability (70%); Shortage of staff (61.5%); Time-consuming to use (30%)

Quality Assessment of Studies

The quality assessment of the included studies was guided by a validated checklist for evaluating studies with diverse designs by Sirriyeh and colleagues [26]. Each eligible study was assessed against 14 points on the checklist if the study was either qualitative or quantitative and 16 points on the checklist if it used mixed methods (Table 3). A percentage score was calculated for each paper to measure quality. Studies that scored less than 50%, 50% to 70%, and greater than 70% were considered poor quality, moderate, and high quality, respectively. All the papers included in this review were of high quality, ranging from 71.4% [36,56] to 92.9% [34,57].

**Table 3:** Quality assessment for papers using a validated checklist Reference: [27]

Criteria	Anokye et al. 2019 [53]	Ayebare et al. 2020 [36]	Ayehubizu et al. 2022 [41]	Bazirete et al. 2017 [34]	Sama et al. 2017 [37]	Bedada et al. 2020 [39]	Hagos et al. 2020 [48]	Brits et al. 2020 [45]	Zelellw and Tegegne 2018 [12]	Mezmur et al. 2017 [47]	Tilahun et al. 2021 [49]	Okokon et al. 2014 [38]	Markos et al. 2020 [43]	Haile et al. 2020 [10]	Negash and Alelgn 2022 [50]	Lumadi 2017 [35]	Konlan et al. 2016 [54]	Melese et al. 2020 [42]	Mukisa et al. 2019 [51]	Wakgari et al. 2015 [32]	Wakgari et al. 2015 [9]	Yang et al. 2017 [46]	Asibong et al. 2014 [53]	Zelellw and Tegegne 2018 [33]	Hailu et al. 2018 [52]	Gebreslassie et al. 2019 [44]	Jagau et al. 2017 [40]	Yisma et al. 2013 [31]	
Explicit theoretical framework	0	0	1	3	0	0	0	0	0	0	0	0	0	0	0	0	0	0	0	0	0	0	0	0	0	0	2	0	
Statement of aims/objectives in main body of report	3	3	3	3	3	3	3	3	3	3	3	3	3	3	3	3	3	3	3	3	3	3	3	3	3	3	3	3	
Clear description of research setting	3	3	3	3	3	3	3	3	3	3	3	3	3	3	3	3	3	3	3	3	3	3	3	3	3	3	3	3	
Evidence of sample size considered in terms of analysis	3	2	3	3	2	3	3	2	3	3	3	3	3	3	3	3	3	3	3	3	3	3	3	3	3	3	3	3	
Representative sample of target group of a reasonable size	3	3	3	3	3	3	3	2	3	3	3	3	3	3	3	3	3	3	3	3	3	3	3	3	3	3	3	3	
Description of procedure for data collection	3	3	3	3	3	3	3	3	3	3	3	3	3	3	3	3	3	3	3	3	3	3	3	3	3	3	3	3	
Rationale for choice of data collection tool(s)	3	2	3	2	3	3	3	2	3	3	3	3	3	3	3	3	3	3	3	3	3	3	3	3	3	3	3	3	
Detailed recruitment data	0	2	3	3	2	3	3	2	2	3	3	3	3	2	3	3	2	3	3	3	3	3	2	3	3	3	2	3	
Statistical assessment of reliability and validity of measurement tool(s) (Quantitative only)	0	0	3	3	3	2	3	2	1	1	2	3	3	3	3	3	3	3	0	3	3	0	3	3	1	3	2	3	
Fit between stated research question and method of data collection (Quantitative only)	3	0	0	2	2	2	3	3	2	2	3	2	3	3	3		3	3	3	3	3	0	3	3	3	3	3	3	
Fit between stated research question and format and content of data collection tool e.g. interview schedule (Qualitative only)	0	3	0	0	0	0	0	0	2	0	0	0		0	0	3	0	0	0	0	0	3	0	0		0			
Fit between research question and method of analysis (Quantitative only)	3		3	3	3	3	3	2	2	3	3	3	3	3	3	0	3	3	2	3	3	0	3	3	3	2	3	3	
Good justification for analytic method selected	3	3	3	3	2	3	2	3	2	3	3	3	3	2	3	3	3	3	3	3	3	3	3	3	3	2	3	3	
Assessment of reliability of analytic process (Qualitative only)	0	3		3			3	3	0	0	0		3			3	0	0	0	0		1	3						
Evidence of user involvement in design	0	0	0	0	0	0	0	0	0	0	0	0	0	0	0	0	0	0	0	0	0	0	0	0					
Strengths and limitations critically discussed	3	3	1	2	2	3	3	3	3	3	3	3	3	3	3	2	3	2	2	1	2	3	2	2	2	2	2	2	
Total	30	30	32	39	31	34	38	33	32	33	35	35	39	34	36	35	35	35	31	34	35	31	37	35	33	33	35	35	
Percentage	71.4	71.4	76.2	92.9	73.8	81	90.5	78.6	76.2	78.6	83.3	83.3	92.9	81	85.7	83.3	83.3	83.3	73.8	81	83.3	73.8	88.1	83.3	78.6	78.6	83.3	83.3	

Consolidated Framework for Implementation Research

CFIR which provides a realistic structure of approaching multilevel constructs [23] and providing a comprehensive assessment of barriers/gaps and facilitators of service uptake within five domains which include: 1) intervention characteristics that influence a given intervention, 2) outer settings that includes external features that could affect utilization of the partograph in labor monitoring, 3) inner setting that includes features within facilities that could influence the use of the partograph, 4) characteristics of individuals like knowledge and beliefs of care givers involved in using the partograph in hospitals that might influence the utilization of the partograph, and 5) implementation process that highlights procedures that might influence the utilization of the partograph for labor monitoring. The CFIR framework thus helps facilitate implementers identifying and addressing context-specific barriers/gaps that could compromise the practicability, functionality, adaptability, adoptability, and scalability of effective health interventions. The CFIR framework has previously been utilized in Sub-Saharan Africa to assess barriers and motivators to private hospitals’ engagement in tuberculosis care in Uganda [58,59], as well as interpret research findings in a review by Leonard and colleagues that aimed at identifying, assessing, and synthesizing facilitators and barriers to sustainably implement evidence-based health innovations in low- and middle-income countries [60]. The application of CFIR to understand the gaps, opportunities, and challenges associated with partograph utilization is crucial in guiding future implementation of similar interventions in maternity care and health care utilization.

Results

Search Results

The bibliographical database search results, screening, exclusion, and final inclusion in the study are presented in Figure 1. The studies described and identified partograph utilization opportunities and challenges in Sub-Saharan Africa, reported in seven countries where the studies were carried out (Ghana, Ethiopia, Uganda, South Africa, Nigeria, Cameroon, and Rwanda). All studies included in this review and their characteristics are shown in Table 2.

Consolidated Framework for Implementation Research

The opportunities and challenges of partograph utilization in Sub-Saharan Africa were categorized under the five domains of the CFIR framework, namely, the intervention characteristics, outer setting, inner setting, characteristics of the individuals, and the process of implementation. Of the 39 CFIR constructs assessed, 10 were relevant to the context of this review, as shown in Table 4.

**Table 4:** CFIR constructs and their related opportunities or challenges to partograph utilization in labor care monitoring in Sub-Saharan Africa CFIR: Consolidated Framework for Implementation Research; OB: obstetric/obstetrician; ANC: antenatal care; EMOC: emergency obstetric care; HR: human resources

CFIR Domain	CFIR construct	Opportunity or challenge	Explanation for opportunities and challenges
Intervention characteristics	Evidence strength and quality	Opportunity	Partograph has the potential to reduce the risk of maternal and fetal mortality and morbidity
	Complexity	Challenge	The partograph was reported to be a complex tool to use for labor care monitoring
	Relative advantage	Opportunity	The partograph facilitates the passing of health information among midwives when handing over shifts
	Design quality and packaging	Challenge	Utilization of the partograph is considered time-consuming due to too much detail required to fill for the obstetric caregivers
Outer setting	External policy and incentives	Challenge	Lack of motivation among staff to use the partograph for labor monitoring
			Different monitoring tools other than the partograph like clinical records, monitoring charts, piece of papers
Inner setting	Structure characteristics	Opportunity	The availability of the partograph sheets in the health facilities
		Challenge	Unavailability of the partograph in health facilities hinders its utilization
			Shortage of staff in hospitals increase the work overload on obstetric caregivers to monitor labor using the partograph
	Culture	Opportunity	Supervising obstetric care providers enhances the utilization
			Positive feedback about the usage of the partograph increases motivation and performance
		Challenge	Lack of encouragement and praise when the utilization of the partograph is done correctly demotivates the utilization
			Negative feedback was interpreted as a sign of being ungrateful which demotivates midwives from using the partograph
Characteristics of individuals	Knowledge and beliefs about the intervention	Opportunity	Training facilitates partograph utilization in labor monitoring
		Challenge	Lack of training or knowledge creates incompetency and knowledge gaps
			Pregnant women’s unwillingness to be examined frequently
	Individual stage of change	Challenge	Negligence and lack of commitment among obstetric care workers in form of careless partograph completion
		Opportunity	Emotional consequences like trauma, sleeping difficulties and stress regarding non-use propels midwives to use the partograph
The process of implementation	Executing	Opportunity	Policy or standard protocol for guidance on the use of partograph
			Mentorship

Intervention Characteristics

Nine studies conducted in Ethiopia, Cameroon, Nigeria, South Africa, Ghana, and Uganda [37,38,40,45,46,52,55,61,62] highlight the ability of the partograph to reduce the risk of maternal and fetal mortality and morbidity [61]. This is through facilitating the detection of prolonged labor [55,61,62], obstructed labor [55], poor labor progress [55], inefficient uterine contraction [55], suspected fetal distress [55], the need for labor augmentation [55], and postpartum hemorrhage [61]. In addition, the partograph helps in distinguishing abnormal from normal labor progress [46], has fewer non-asphyxiated birth outcomes [56], facilitates early referral to specialized health facilities [62], and increases the efficiency of those attending to women in labor [37]; thus, potentially improving labor and delivery outcomes [40].

Two qualitative investigations in Uganda [46,51], carried out to determine the level of partograph completion and to explore current practices, challenges, and opportunities of partograph utilization, respectively, found that the partograph was important in facilitating the passing of labor monitoring information among midwives when handing over shifts and between midwives and doctors during reporting, which enhances decision-making regarding labor progress among midwives and doctors [46].

However, several studies [49,51,54,55,62,63] have documented that the utilization of the partograph is time-consuming because too many details are required to be filled by obstetric caregivers, alongside other labor monitoring notes in patient files, creating extra workload on already overloaded midwives [39,43,46,51,54]. Additionally, a cross-sectional study conducted to assess knowledge of the partograph and its associated factors among obstetric care providers in Ethiopia found the partograph a complex tool to use for labor monitoring [9], which explains why some obstetric care providers didn’t use the tool.

Outer Setting

The presence of different monitoring tools other than the partograph, such as clinical patient notes, monitoring charts [42], and cardiotocographs (CTG) [51], was reported as a key challenge to utilizing the partograph in labor monitoring in Ethiopia and Uganda. These created duplicated documentation, consuming time for midwives. Lack of motivation among staff to use the partograph for labor monitoring was reported in Ethiopia [61], where midwives and other obstetric caregivers often relaxed and got demoralized from continuing to consistently fill and use the partograph.

Inner Setting

Three studies in Ethiopia and Ghana [39,41,54] documented that the availability of partographs in the delivery rooms facilitated utilization among healthcare providers. Health care providers in Ethiopia who had partographs readily available in their facilities were four times more likely to monitor labor routinely using them than those in facilities where the partographs were not readily available [39,41]. Indeed, the unavailability of the partograph was reported to be the main hindrance to its utilization in labor monitoring in Ethiopia [32,42,43].

A qualitative descriptive study aiming at exploring the perceptions of midwives regarding audit and feedback on partograph use in Limpopo Province in South Africa found that positive feedback increased motivation and performance. Negative feedback was a sign of being ungrateful and demotivated and caused dissatisfaction among midwives. Lack of encouragement and praise when the partograph was filled correctly was cited as one of the challenges that discouraged usability. Emphasis was mostly placed on negative aspects when the partograph was not filled correctly, and this demotivated midwives and other healthcare providers to complete and consistently use the partograph [35].

In central Ethiopia, obstetric care providers who had routine presence of supervisors were four times more likely to use the partograph compared to those whose supervisors were not present [39]. Skilled labor attendants who had supportive supervision were four and a half times more likely to utilize the partograph for labor monitoring compared to their counterparts who did not receive support supervision [50]. A cross-sectional study conducted in Addis Ababa reported that midwives who received supportive supervision once a year were three times more likely to consistently use the partograph, while those who received supervision twice a year were four times more likely to use the partograph than those who did not. Even better, those who received supportive supervision four times a year were 18 times more likely to use the partograph than those who did not get supervised at all [10]. On the other hand, the absence of managerial or responsible bodies to carry out supervisory obligations in hospitals has been reported by four studies in Ethiopia [10,39,41,43] as a hindrance to effective utilization of the partograph in labor monitoring.

Studies in Ghana, South Africa, Ethiopia, Nigeria, and Cameroon [9,35,37,45,49,55] noted a shortage of staff in hospitals, with increased work overload on the existing few health workers hindering proper monitoring of pregnant women in labor. Skilled birth attendants prioritized emergencies and attending to patients in busy clinics and delivery rooms rather than filling and writing down all observations on the partograph [40,45], resulting in nonuse or misuse of the partograph.

Characteristics of the Individuals

Fourteen studies [9,32,34,39,41,43,44,47,48,50,52,54,61,64] emphasized the role of training as a facilitator for partograph utilization in labor monitoring in Sub-Saharan Africa. Several studies reported that adequately trained obstetric caregivers had over 15.46 [41], 7.06 [10], 5.49, 3.94 [39], 2.86, 2.4 [10], 2 [47], and 1.9 [50] higher odds, respectively, for using the partograph properly than those who did not have adequate training. Lack of adequate training or knowledge created incompetency, knowledge gaps, lack of confidence, and bias among health care providers to confidently use the tool, which were cited as a main barrier to partograph utilization in Sub-Saharan Africa [10,34,37,39,40,43,45,47,49,55,61,62].

A positive attitude toward partograph utilization was documented as one of the components that facilitated its use. Nine studies [9,10,39,41-43,45,47,52] conducted in Ethiopia and South Africa clearly spelled out that obstetric caregivers who had a favorable or positive attitude toward using the partograph had over 2.9 [41,43,47], 2.48 [39], 2.35, and 1.8 [10], respectively, higher odds of using the partograph compared to their counterparts who had a negative attitude [47,61].

A sequential explanatory mixed methods study (conducted following the audit of poor obstetric outcomes) to explore fetal heart rate monitoring practices among health workers at a public hospital in Northern Uganda documented regret and emotional consequences like trauma, sleeping difficulties, and stress. Fear of these consequences related to partograph non-use or misuse encouraged the utilization of the partograph to monitor women during labor [36]. A cross-sectional study in Ethiopia explored the use and barriers of partograph use among obstetric caregivers in Northwest Ethiopia. Negligence, carelessness, perceived boredom, and work overload-related fatigue were reported as major barriers in this region [61]. Interestingly, exhaustion resulting from inadequate staffing levels coupled with large numbers of patients in busy units resulted in retrospective plotting/filling of the partograph after childbirth to simply fulfill hospital protocol requirements, rather than using it to make labor decisions [36,45,46]. The lack of commitment among obstetric care providers was also reported in Ethiopia [10,39,41]. Obstetric care providers who lacked commitment to partograph use were 68% less likely to utilize the partograph for monitoring mothers in labor compared to those who were committed to utilizing the partograph (AOR = 0.32, 95% CI: 0.16, 0.63) [39].

Another patient-related challenge that was cited was the pregnant women’s unwillingness to be examined frequently, while others would choose to go home when told to ambulate, and by the time they return to the hospital, they are already in the second stage of labor, making the process of monitoring incomplete [36].

Process of Implementation

A facility-based cross-sectional study in West Shoa Zone, Central Ethiopia, to assess the level of partograph utilization and associated factors reported that obstetric care providers that had a policy or standard protocol for guidance on the use of partographs were five times more likely to use the partograph than those who did not have [39]. Continuous mentorship was highlighted as a key determinant in supporting, teaching, discussing, and empowering midwives and other users in sustained labor monitoring. A cross-sectional study in Ethiopia that assessed utilization and predictors of partograph use found that midwives who were mentored by an expert were three times more likely to use partographs than those who were not (AOR = 3.0; 95% CI: 1.8, 5.4) [10].

Discussion

This review aimed at synthesizing and documenting the current published evidence regarding the opportunities and challenges, or gaps, of partograph utilization in labor monitoring in Sub-Saharan Africa in the past decade, that is, overlapping the transition from the long-used partograph to the newly recommended labor care guide. We used CFIR to analyze the gaps, challenges, and opportunities associated with partograph utilization and used the lessons learned to guide the implementation of similar interventions in maternity care, such as the new labor care guide. This review was guided by two main questions: 1) What facilitates the utilization of the partograph in labor monitoring in Sub-Saharan Africa? and 2) What hinders the utilization of the partograph in labor monitoring in Sub-Saharan Africa? Twenty-eight high-quality studies met our eight-point inclusion criteria and were analyzed for review.

Opportunities identified by our review included 1) the partograph’s uncontested potential to reduce maternal and neonatal mortality and morbidity facilitated by good support supervision, formal and on-the-job training, active mentorships, ongoing supervision, and availability of enabling policies, standards, and protocols on partograph use; 2) partographs’ availability in health facilities, which facilitated handover and transfer of labor information between obstetric care providers and work shifts.

On the other hand the gaps and challenges identified included: 1) lack of or inadequate training, tool complexity, too much detail required to fill the non-stand-alone partograph resulting in time consumption during labor monitoring, 2) availability of different and or supplementary monitoring tools other than the partograph which often fatigued and biased users, 3) lack of motivation and feedback, 4) unavailability of the partograph in some health facilities, 5) shortage of staff in busy facilities, 6) lack of support supervision, 7) lack of protocols, 8) inappropriate motivation (including lack of encouragement, praises nor reward for consistent use) leading to 9) lack of commitment, negative attitude, negligence, careless partograph completion or non-use. These challenges present major obstacles that frustrate optimal utilization and its associated outcomes on labor monitoring [47].

This review systematically used the trusted CFIR and presents a synopsis of gaps, challenges, and barriers hindering partograph utilization and used the identified opportunities and lessons learned to present a sound platform for designing and developing suitable implementation strategies for the introduction, adoption, adaptation, and scale-up of the new labor care guide. Given the criticality of intrapartum monitoring for women and their unborn babies and its potential to improve labor and delivery outcomes, persistence of these challenges and gaps experienced by obstetric care providers using the partograph over the past 50 years should not be overlooked but rather used as lessons to guide practice using the new labor care guide. Whereas it is understandable that some of these challenges are structural and organizational, like a shortage of staff and unavailability of the partograph, which may be beyond individuals’ control, it is important to ensure that efforts are put in place to lessen the impact of these challenges on labor monitoring in Sub-Saharan Africa. Besides, many studies have widely observed high incidences of obstructed labor and its associated complications and deaths, mainly attributed to suboptimal utilization of the partograph within fairly staffed facilities, with largely available partographs in the patient files [9,10,13]. This may partly be due to the partograph’s subjective variations and its assumption that all women progress at the same rate, which may affect the intervention rate [12], but also a lack of commitment, support supervision, guiding protocols, motivation, negative attitude, and negligence as reported in many reviewed studies [9,10,34,39,41,43,44,46,47,50,52,54,61].

Several reviews have also documented these similar barriers regarding partograph utilization in labor monitoring. A realist review by Bedwell and colleagues shows that although the partograph has been accepted, evidence shows that it has not been utilized as expected in real practice, thus failing to achieve its intended outcomes [14]. Noteworthy, this review found that healthcare providers’ positive attitude and support to use the partograph alone did not translate into practice. Ollerhead and Osrin, on the other hand, have argued that the partograph can only improve maternal care outcomes in settings running an effective maternity care system [15], contrary to most healthcare systems in Sub-Saharan Africa characterized by a high patient-doctor ratio due to staff shortages [65], poor infrastructure in terms of bed capacity (1.3 hospital beds per 1000 people) as compared to 2.1 in Latin America and 6.1 in Europe.

The WHO discouraged the use of ineffective practices such as the partograph [66] in labor and childbirth in an effort to avert maternal and perinatal deaths and instead has called for the adoption and evaluation of adaptable and context-specific health solutions to improve labor monitoring and promote better health outcomes [67]. Identifying and scaling up such interventions to improve the quality of healthcare and outcomes in pregnancy, childbirth, and the immediate postnatal period has great potential to prevent a substantial number of annual 823,000 stillbirths, 1,145,000 neonatal deaths, and 166,000 maternal deaths in the 75 highest burden countries [68-70]. The WHO recommended the adoption of the labor care guide that aims to support good-quality, evidence-based, respectful care during labor and childbirth, irrespective of the setting or level of health care. Although the labor care guide is not yet introduced for routine use as a decision-making tool in many maternity care settings with high rates of prolonged/obstructed labor and persistently high maternal and perinatal mortality [70,71], understanding these gaps and opportunities faced by the partograph may present potential lessons to guide adoption and scale-up of the labor care guide. These potential lessons are necessary in settings like Uganda, where the labor care guide is in the initial stages of introduction and use in routine labor monitoring [71,72] and therefore will guide the development of suitable implementation strategies for a successful rollout.

Contribution of this review

This review used CFIR principles to synthesize the gaps/challenges and opportunities and used them to present potential lessons to guide the adoption of the labor care guide in Sub-Saharan Africa. It contributes to the body of knowledge in the field of intrapartum care, proposes potential lessons, and sets a firm ground to galvanize the introduction of the labor care guide as a decision-making tool while monitoring labor. The results from this review will help stakeholders, policymakers, researchers, and ministries of health in Sub-Saharan Africa and other similar low-resource settings to advance safe motherhood interventions to improve labor and delivery outcomes.

Strengths and limitations

Our study is the first to document the opportunities and challenges and present lessons learned from partograph utilization in labor monitoring in Sub-Saharan Africa and similar settings. Evidence from this study contributes to the body of knowledge for policymakers and implementers about what needs to be borne in mind to effectively and optimally implement other labor monitoring tools in Sub-Saharan settings where there are persistent maternal and perinatal deaths. Our review is based on implementation science principles, including evidence-based practice gap identification using a theoretical framework like CFIR. Understanding the opportunities and challenges influencing the utilization of the partograph using the CFIR framework offers a practical way of addressing the identified challenges and using lessons learned to guide the design of suitable implementation strategies for evidence-based interventions.

Nevertheless, our review is not without limitations. First, the review focused on challenges and opportunities reported exclusively in Sub-Saharan Africa. Secondly, despite the extensive search, only 28 studies were deemed to be of high quality and reported on partograph utilization as guided by a validated checklist for evaluating studies with diverse designs [26]. Therefore, the recommendations made by the researchers and the interpretation of the study findings should be done with this regard. Last, this review only included studies published in the English language as understood by the investigators, which could have limited the diversity of findings presented in other languages.

## Conclusions

Despite its benefits in labor monitoring, the persistent challenges continue to limit the partograph’s potential to optimize and/or foster effective labor monitoring. Rethinking the partograph utilization, given its gaps and shortcomings, should be given attention. The adoption of the alternative recommended mechanisms, such as the labor care guide, may revolutionize labor monitoring in Sub-Saharan Africa, whose unique challenges may not be a fit for all. Therefore, keen attention is needed to clearly think through the best implementation strategies in order to avoid the same mistakes hindering the partograph’s optimal use.
